# Spike- and nucleocapsid-based gold colloid assay toward the development of an adhesive bandage for rapid SARS-CoV-2 immune response detection and screening

**DOI:** 10.1038/s41378-023-00554-8

**Published:** 2023-06-20

**Authors:** Imen Boumar, Muhammedin Deliorman, Pavithra Sukumar, Mohammad A. Qasaimeh

**Affiliations:** 1 Division of Engineering, New York University Abu Dhabi (NYUAD), Abu Dhabi, UAE; 2 NYU Tandon School of Engineering, New York University, New York, USA

**Keywords:** Chemistry, Biosensors, Microfluidics

## Abstract

Immunoglobulin M (IgM) and immunoglobulin G (IgG) antibodies are important biomarkers used for the diagnosis and screening of severe acute respiratory syndrome coronavirus 2 (SARS-CoV-2) infections in both symptomatic and asymptomatic individuals. These antibodies are highly specific to the spike (S) and nucleocapsid (N) proteins of the SARS-CoV-2 virus. This paper outlines the development steps of a novel hybrid (vertical-lateral-vertical) flow assay in the form of a finger-stick point-of-care device, similar to an adhesive bandage, designed for the timely detection and screening of IgM and IgG immune responses to SARS-CoV-2 infections. The assay, comprising a vertically stacked plasma/serum separation membrane, conjugate pad, and detection (readout) zone, utilizes gold nanoparticles (AuNPs) conjugated with SARS-CoV-2 S and N proteins to effectively capture IgM and IgG antibodies from a pinprick (~15 µL) of blood in just one step and provides results of no immune IgM−/IgG−, early immune IgM+/IgG−, active immune IgM+/IgG+ or immune IgM−/IgG+ in a short amount of time (minutes). The adhesive bandage-like construction is an example of the design of rapid, low-cost, disposable, and easy-to-use tests for large-scale detection and screening in households. Furthermore, the bandage can be easily adjusted and optimized to detect different viral infections as they arise by simply selecting appropriate antigens related to pandemics and outbreaks.

## Introduction

Since its first occurrence in Wuhan province of China in December 2019^1,2^, coronavirus disease 2019 (COVID-19) has reached, as of 10 June 2023, a grim landmark of over 767 million confirmed cases worldwide, including 6.9 million deaths^3^. However, the incidence of disease caused by severe acute respiratory syndrome coronavirus 2 (SARS-CoV-2) infection is believed to be underdiagnosed and thus underreported. For example, in the United States alone, it is estimated that for every reported case, there are 3–20 undiagnosed cases^4^. Clearly, the true scale of COVID-19 is bounded by low PCR test rates, which is primarily due to the availability of key supplies and low adoption rates of new viral gene targets^5^.

With growing evidence of the waning effectiveness of current vaccines over time^6^, along with the high transmissibility of emerging SARS-CoV-2 variants^7^, increased testing is crucial to prevent further infections. In fact, it was reported that high testing and screening rates would be more effective in reducing SARS-CoV-2 infection rates than revaccination campaigns^8^. In particular, in that study, it was highlighted that until booster shots are administered to more than 1% of the total population per day, detecting and screening asymptomatic individuals (i.e., infected individuals with no symptoms) is paramount for reducing the overall number of COVID-19 cases^8^. Asymptomatic individuals are a significant source of COVID-19 transmission because their viral loads in the upper respiratory tract are comparable to those of symptomatic individuals^9,10^. This implies that infectiousness may peak before or at the onset of symptoms^11^. Therefore, there is a continuous need to explore rapid, inexpensive, and practical methods for the early detection and screening of COVID-19 cases, especially among asymptomatic individuals. While this study focused on COVID-19 applications, it is important to note that the developed technology and related discussions are also applicable to other viral/bacterial infections.

At the time of SARS-CoV-2 infection and during its different stages, the immune response is the first line of defense and involves the production of immunoglobulin M (IgM) and immunoglobulin G (IgG) antibodies in the blood^12,13^. These antibodies inhibit the viral load by binding to the spike (S) and nucleocapsid (N) proteins of SARS-CoV-2^14,15^. In symptomatic individuals, IgM antibodies appear in the early stages of viral infection and are therefore an important indicator of the peak infection period. IgG antibodies, on the other hand, replace IgM antibodies after the onset of symptoms and are essential for long-term immunity and immunological memory^16,17^. Interestingly, in asymptomatic individuals, the concentration of IgM antibodies is reported to be significantly higher than in healthy subjects and not easily degraded within 7 weeks of infection. Moreover, the concentrations of IgG antibodies are reported to be above normal reference levels and to increase with time during the 7 weeks after infection^18^. Therefore, it becomes important to dynamically monitor IgM and IgG antibodies for efficient diagnosis and screening of SARS-CoV-2 infections^12^ in both symptomatic and asymptomatic individuals^18,19^. To establish this, antibody (serology) tests provide an ideal approach for early detection as well as for determining the percentage of the population that is infected.

Due to the variability in the level of protective immunity among people, a large number of antibody-based detection and screening techniques are available in clinical laboratories, such as agglutination, enzyme immunoassay (EIA), and enzyme-linked immunosorbent assay (ELISA)^20^. Agglutination is a rapid method, taking only 15–20 min, but requires large amounts of antigens for visible agglutination. EIA and ELISA, on the other hand, require the labeling of captured antibodies with fluorescent molecules or enzymes for detection, identification, and quantification. All these techniques can be adapted for high-throughput and full-automation configurations with varying degrees of success. However, the limited number of antibody-antigen pairs that they can handle and the inability to process samples on a large-scale present a hurdle. Moreover, these techniques, although highly specific and sensitive, do not lend themselves to point-of-care applications due to the necessity of equipment and skilled personnel.

Rapid point-of-care antibody tests offer an alternative to laboratory antibody tests. Among them, lateral flow assays (LFAs), with working mechanisms similar to those of standard pregnancy tests^21^, are a notable example that has been widely developed for colloid-based capture and detection of SARS-CoV-2 IgM and/or IgG antibodies from blood^22^. However, LFAs have limitations, among which saturating the test (capture) lines with unbound (“free”) antibodies is particularly important. Accordingly, in a typical LFA, a plasma/serum sample containing an unknown concentration of target antibodies is allowed to sequentially pass through the detection bands, which are activated with a fixed amount of corresponding capture antibodies. Ideally, the amount of the capture antibodies should be proportional to the concentration of the target antibodies in the plasma/serum. However, in cases where the concentration of the target antibodies is very high, the detection bands become saturated and unable to bind to all the target antibodies present. As a result, the signal detected by LFA plateaus or even decreases with increasing concentrations of the target antibodies, leading to a false-negative result^23^. This phenomenon is known as the “hook effect” because the graph of the LFA signal versus target antibody concentration appears to turn downward like a fishhook. Thus, to overcome the “hook effect”, it is necessary to dilute the plasma sample in LFAs to tolerate the capture of high concentrations of target antibodies. Other limitations of LFAs include the use of “chase” buffer, risk of cross-contamination, and limited ability to provide simultaneous results for multiple target analytes^24^. In contrast, colloid-based vertical flow assays (VFAs) prevent the hook effect by sequentially delivering excess antibodies directly to the respective test zones. Therefore, VFAs are emerging as alternatives to LFAs for detecting antibodies in various viral infections^25,26^. However, it is worth noting that most VFAs are fabricated inside cassettes, which require additional blood handling via capillary tubes.

**Fig. 1:**
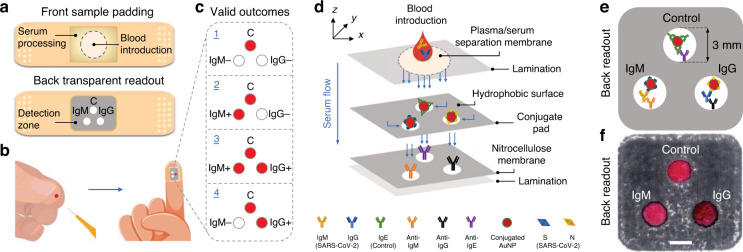
Adhesive bandage-based on a hybrid (vertical-lateral-vertical) flow assay (HFA) for the capture and detection of SARS-CoV-2 IgM and IgG antibodies. **a** Schematic of the finger-stick point-of-care device (adhesive bandage), along with its **b** in-home use and **c** valid outcomes confirming 1: negative (IgM−/IgG−) or 2–4: positive (IgM+/IgG−, IgM+/IgG+, IgM−/IgG+, respectively) for SARS‐CoV‐2 infection. C represents a positive control spot for the valid outcomes. **d** Schematic of the HFA’s bioactive layers and their working mechanism for gold colloid-based capture and detection of IgM and IgG antibodies from a pinprick (~15 µL) of blood. Following blood introduction, the plasma/serum (separated from blood cells using a filter membrane) flows first into the hydrophilic conjugate spots and then into hydrophilic nitrocellulose detection spots in vertical-lateral-vertical directions (blue arrows). The conjugate spots contain dry AuNP-S and AuNP-N bioconjugates for the capture of IgM and IgG antibodies, together with dry AuNP-IgE complexes to serve as controls. Hydrophilic and hydrophobic regions in the conjugate pad and nitrocellulose membrane are represented in white and gray, respectively. Each spot in the detection zone of the nitrocellulose membrane contains binding surfaces coated with anti-IgM and anti-IgG secondary (capture) antibodies specific to targeted IgM and IgG. In the control spot, anti-IgE antibodies serve to capture only IgE antibodies. **e** Schematic shows the back readout of the HFA when a mixture of IgM and IgG antibodies was introduced to the conjugate pad. **f** Micrograph shows the back readout of an HFA with active immune (IgM+/IgG+) outcome, thus proving the feasibility of the adhesive bandage. Scale bar: 3 mm

The work described in this study presents the development steps of a novel hybrid (vertical-lateral-vertical) flow assay, named HFA, in a user-friendly adhesive bandage-like device. The HFA takes advantage of the working principles of both lateral and vertical flow assays, making it suitable for in-home use. The HFA device utilizes SARS-CoV-2-specific S and N proteins within a single test to enhance sensitivity, as shown in Fig. 1. The device is designed to colloid-capture SARS-CoV-2-specific IgM and IgG antibodies from a small pinprick of blood (~15 µL) in just one step, making it a rapid detection and screening tool for assessing the immune response. Furthermore, the device can be easily adjusted and optimized to capture and detect different viral infections by selecting appropriate antigens related to any future pandemics and outbreaks.

## Results

### Design and working principle of the HFA

The HFA is designed in the form of an adhesive bandage for user-friendly in-home use (Fig. 1a). It consists of a front sample padding where blood is introduced and a back transparent readout where the detection zone is located. When a pinprick of blood (~15 µL) is introduced to the pad (Fig. 1b), a red color appears at the control spot for all valid results to confirm whether the sample is negative (IgM−/IgG−) or positive (IgM+/IgG−, IgM+/IgG+, IgM−/IgG+) for SARS‐CoV‐2 infection (Fig. 1c).

The blood processing area of the bandage (as depicted in Fig. 1a) consists of a plasma/serum separation membrane, a conjugate pad, and a nitrocellulose membrane, all vertically stacked on top of each other, with each component serving a specific function (Fig. 1d). After blood introduction, the separation membrane (filter paper) with ~6 µm pore size retains blood cells with ~97% efficiency while allowing plasma/serum to flow vertically into the conjugate pad (Fig. 1d, top panel, Supplementary Fig. S1, Materials and Methods). The SARS-CoV-2 IgM and/or IgG antibodies then interact with dried gold nanoparticles (AuNPs) residing in the controllable spots of the conjugate pad (Fig. 1d, middle panel).

To prevent the competitive nonspecific binding of other proteins in plasma/serum, the AuNPs used in the assay are functionalized with specific SARS-CoV-2 S and N proteins that target IgM and IgG antibodies, creating “lock and key” interactions. For control measurements, AuNPs are functionalized with human recombinant IgE protein, which is known for its specific binding to allergenic antigens and triggering of inflammatory responses through the release of histamine and other inflammatory substances and has been extensively studied with antibody Fab fragments^27^. This allows the AuNP-IgE complexes to serve as unique probes for control purposes, as they do not preferentially bind to IgM and IgG antibodies, thus minimizing cross-reactivity. Moreover, for uniform horizontal flow and controlled delivery of plasma/serum onto AuNPs, the conjugate pad is chemically modified to contain three hydrophilic spots (~3 mm diameter) that are surrounded by hydrophobic barriers. One spot contains AuNP-IgE bioconjugates for the validation of the assay (control), while the other two spots contain AuNP-S and AuNP-N bioconjugates for capturing IgM and IgG antibodies. Following interaction with AuNPs, the antibody-AuNP complexes then flow vertically to the detection zone by capillary forces, ensuring controlled delivery for accurate results.

The nitrocellulose membrane forms the detection zone of the assay (Fig. 1d, bottom panel). It is compartmentalized to form three hydrophilic spots surrounded by hydrophobic barriers. To ensure optimal analyte delivery and minimize cross-reactivity, the spot pattern on the nitrocellulose membrane (printed using a wax printer, see Materials and Methods) is designed to match the spot pattern on the conjugate pad. Here, the binding surfaces of the spots are coated with anti-IgE, anti-IgM, and anti-IgG secondary antibodies, which specifically capture and detect IgE (control), IgM, and IgG antibodies, respectively (Fig. 1e). The validation of results occurs when any of the antibody spots in the detection zone show a visible reaction (i.e., production of a red color) along with the control spot (Fig. 1f).

### Characterization of AuNP bioconjugates

**Fig. 2:**
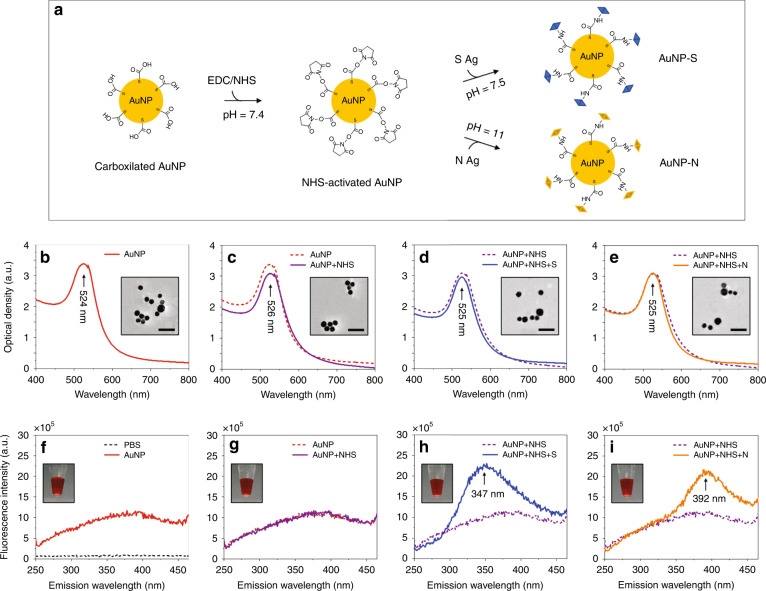
Bioactivation of the AuNPs with SARS-CoV-2 S and N proteins and their characterization. **a** Schematic illustrates the sequential steps involved in the covalent binding of S and N proteins to carboxylated AuNPs via EDC/NHS chemistry. Buffer solutions with pH 7.5 and 11 were utilized to achieve stable coupling of AuNP-S and AuNP-N bioconjugates, respectively. **b**–**e** Optical density measurements and TEM images (insets) confirmed the stability of the solutions, showing monodispersed AuNPs after chemisorption of each functional group to each layer of activated AuNP surfaces. Scale bars: 200 nm. **f**–**i** Intrinsic fluorescence measurements of the S and N proteins, with excitation at 240 nm, further validated their successful chemisorption to AuNPs via EDC/NHS chemistry. Insets: the color of AuNP solutions remained unchanged as a function of surface activation, thus indicating their stability under optimized conditions

The first development step in our work focused on optimizing the binding of SARS-CoV-2 S and N proteins to AuNP surfaces to enhance the specific capture of IgM and IgG antibodies. To achieve this, carboxylated AuNPs (~40 nm diameter) were modified using EDC/NHS chemistry (see Materials and Methods) to convert the surface carboxyl (−COOH) groups into more amino-reactive −NHS esters that would efficiently cross-link the S and N proteins through their primary amines (Fig. 2a). The cross-linking reaction resulted in the formation of intermediate covalent amide bonds between the carboxyl groups and primary amines (catalyzed by EDC), which exhibited increased S and N coupling stability at pH 7.5 and 11, respectively.

Effective step-by-step characterization of the chemically modified AuNP surfaces was performed using UV‒Vis and fluorescence spectroscopies. First, chemisorption of each functional group to each layer of activated AuNP surfaces was confirmed by UV‒Vis spectroscopy (Fig. 2b–e). The strong absorption peak initially observed at ~524 nm corresponded to a typical plasmonic band of 40 nm diameter carboxylated AuNPs^28^. When AuNPs were assembled through the addition of EDC/NHS, the absorbance peak shifted to ~526 nm due to a change in the local refractive index at the AuNP surface, which indicated NHS ester formation. To minimize AuNP aggregation, the reaction was carried out in potassium carbonate solution at pH = 11. Subsequently, S and N proteins were introduced at 1 mg mL^−1^ concentrations, and the pH of the solutions was readjusted to 7.5 and 11 for AuNP-S and AuNP-N bioconjugation, respectively. Successful AuNP-S and AuNP-N functionalization was verified by a 1 nm shift in the final absorption peak (from ~526 nm to ~525 nm), which indicated efficient conjugation of NHS esters to primary amines^28^. Importantly, after each chemical step, the absorbance bandwidths of AuNPs did not broaden, which indicated stable (monodispersed) solutions. This finding agreed with the TEM images, which showed no signs of aggregation (insets in Fig. 2b–e). Similar results were also obtained for the control AuNP-IgE bioconjugates (Supplementary Fig. S2).

Next, the chemisorption of S and N proteins to NHS-activated AuNPs was further validated by measuring their intrinsic fluorescence with fluorescence spectroscopy (Fig. 2f–i). Among the fluorophores found in proteins, tryptophan is reported to be the dominant source of intrinsic protein fluorescence, with its indole group responsible for the absorbance at ~280 nm and emission at ~350 nm^29^. Therefore, during fluorescence measurements, AuNP-S and AuNP-N bioconjugates were excited at 240 nm, and their emission was recorded between 250–450 nm. When excited, the emission of S and N proteins did not interfere with the plasmonic emission of AuNPs, which was ~423 nm when excited at 308 nm^30^. Thus, compared to those of carboxylated and NHS-activated AuNPs in Fig. 2f, g, the emission peaks observed at ~347 nm and ~392 nm in Fig. 2h, i were indicative of the successful chemisorption of S and N proteins, respectively. It should be noted that the emission wavelength of tryptophan is greatly affected by the polarity of its local environment, hydrogen bonding, and other noncovalent interactions, resulting in differences of over 40 nm in peak wavelength^31^. Therefore, the differences in the emission peak wavelengths of the S and N proteins were likely due to the differences in the pH of the AuNP-S and AuNP-N solutions (7.5 and 11, respectively).

It is also worth noting that the color of AuNP solutions remained unchanged as a function of surface activation (insets in Fig. 2f–i), which further confirmed their stability under optimized conditions. Moreover, for the follow-up experiments, AuNP-S and AuNP-N bioconjugates were additionally treated with 0.5% (v/v) protein-free blocking buffer to occupy any unused binding sites on the surfaces. This step was designed to minimize the nonspecific binding of IgM and IgG antibodies to the AuNP surfaces.

### Specificity in IgM and IgG detection

**Fig. 3:**
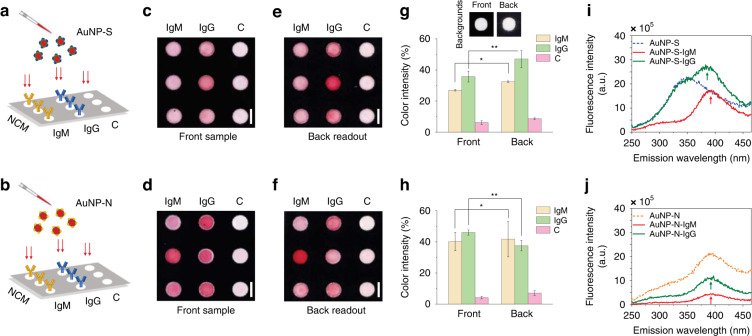
Investigation of the S and N protein specificity toward SARS-CoV-2 IgM and IgG antibodies. **a**, **b** Schematics represent the experimental setup, where IgM and IgG antibodies are first physically adsorbed within the hydrophilic detection spots of nitrocellulose membranes, after which AuNP-S and AuNP-N solutions are directly pipetted onto spots to allow for specific coupling. In both sets of experiments, one line of spots is left untreated as a control (C) to determine the overall nonspecific binding of S and N proteins to nitrocellulose fibers. **c**–**h** Overall, the results revealed that compared to controls, S and N proteins are highly reactive toward coupling with IgM and IgG antibodies. Scale bars: 3 mm. Error bars: mean ± SEM (*n* = 6). * and **: Significantly different at *P* < 0.05 using two-sided Student’s *t* test. The inset in **g** shows the backgrounds for the front sample and back readout used as a reference in color intensity quantification. **i**, **j** The intrinsic fluorescence measurements with excitation at 240 nm further confirmed the binding of IgM and IgG antibodies to S and N proteins with emission peaks measured at ~347 nm (green arrow) and ~392 nm (black arrow), respectively

After successfully creating stable AuNP-S and AuNP-N bioconjugates, the second development step in our work was the investigation of S and N protein specificity toward SARS-CoV-2 IgM and IgG antibodies using nitrocellulose membranes (Fig. 3). The membranes were first compartmentalized using a wax printer, with 3 × 3 hydrophilic detection spots surrounded by hydrophobic barriers. IgM and IgG antibodies were then physically adsorbed within the 2 × 3 detection spots in pH 7 buffer at 1 mg mL^−1^ concentrations for specificity measurements, while the remaining spots were left untreated as controls to reveal the overall nonspecific binding of S and N proteins to nitrocellulose fibers (Fig. 3a, b). Subsequently, the uncoated surfaces of the fibers within the spots were blocked with 0.5% (v/v) protein-free blocking buffer and 0.05% (v/v) Tween-20. Then, 20 µL of AuNP-S and AuNP-N solutions were pipetted onto spots for coupling. The relative color intensities produced in the front sample and back readout of nitrocellulose spots were analyzed and compared.

The color intensity visible to the naked eye indicated that, compared to controls, S and N proteins exhibited a good affinity toward IgM and IgG antibodies (Fig. 3c–f). The color profiles in the images were also consistent and uniform within both the front sample and back readout sides of the spots, suggesting that IgM and IgG antibodies covered the surface of fibers in an oriented manner (i.e., with two antigen-binding sites pointing toward the sample surface^32^). However, the degree of antibody coverage varied from side to side. For example, the coupling of the S to IgM and IgG dominated the back readout by color intensity increases of ~5% and 11%, respectively (Fig. 3g). The coupling of the N to IgM and IgG resulted in an ~2% increase but an 8% decrease in the back readout color intensity, respectively (Fig. 3h).

The observed behavior was mainly attributed to the highly heterogeneous pore size, pore distribution, and porosity of the nitrocellulose membrane. Along with the viscosity and surface tension characteristics of the fluid medium, these factors are known to affect the capillary flow of antibodies and thus lead to nonhomogeneous dispersion within the spots^33,34^. Moreover, it was also shown that when antibodies are allowed to bind to nitrocellulose fibers in acidic buffers (pH 2 and 3), they become more resistant to removal (wash-off) upon treating the fibers with protein-containing blocking agents (e.g., dry milk, BSA) or nonionic detergents (e.g., Triton X-100, Tween-20) compared to their binding in neutral buffers (pH 7)^35^. Thus, during the treatment of nitrocellulose fibers with Tween-20, physically adsorbed IgM and IgG antibodies may have become susceptible to removal, which could have contributed to their nonuniform distribution within the spots. Clearly, to achieve overall increased sensitivity, further investigation is needed to understand the antibody removal dependency on both the pH and type of buffer used to bind antibodies to nitrocellulose fibers, which can be addressed in future studies.

Nevertheless, the color intensity variations between the spots revealed that S proteins exhibit higher reactivity toward IgG antibodies than IgM antibodies, with combined average color intensities of ~41% ± 4% and 30% ± 1%, respectively. For N proteins, these values were ~42% ± 3% and 41% ± 9%, respectively, showing similar reactivity toward IgM and IgG antibodies. Small but persistent nonspecific adsorption of S and N proteins to nitrocellulose fibers was also visually observed in all control spots (Fig. 3c–f). When these spots were characterized for S and N (Fig. 3g, h, respectively), their occurrences were evident with <10% color intensity increases relative to the untreated front and back backgrounds (inset in Fig. 3g).

In general, nonspecific protein-to-fiber binding is either hydrophobic (i.e., can occur between the hydrophobic portion of the protein and carbon-containing nitrocellulose) or electrostatic (i.e., can occur between dipoles within proteins and dipoles of nitrate esters)^33,36^. When combined with the large surface area of the fibers and the highly heterogeneous pore size, pore distribution, and porosity of nitrocellulose membranes, great opportunities are provided for proteins to nonspecifically adsorb to fiber surfaces and pore walls. Therefore, preventing this adsorption is quite challenging but crucial for developing flow assays with enhanced sensitivity, multiplexing, consistency, and reproducibility. To some extent, nonspecific protein-to-fiber binding is minimized by pretreating the fiber surfaces with protein-containing blocking agents (e.g., BSA)^37^ or by chemically modifying them with protein-repelling molecules (e.g., polyethylene glycol)^38^. Nonionic detergents (e.g., Tween-20 in this study), on the other hand, are commonly used to efficiently saturate nonspecific protein binding sites on nitrocellulose fiber surfaces^39^. However, their blocking ability is partially removed during washing with water or buffer, immediately exposing the unblocked areas to protein adsorption (e.g., S and N proteins in this work).

Moving forward, the binding of IgM and IgG antibodies to S and N proteins was further confirmed using fluorescence spectroscopy. In the experiments, IgM and IgG antibodies (at concentrations of 1 mg mL^−1^) were allowed to interact with AuNP-S and AuNP-N bioconjugates, followed by excitation at 240 nm and recording of emission between 250–450 nm. Compared to the emission peaks of S (~342 nm) and N (~392 nm) proteins (see Fig. 2h, i), the emission peaks observed in Fig. 3i, j confirmed the successful binding of IgM and IgG antibodies to S and N proteins, respectively. Specifically, for S-IgM and S-IgG couplings, the emission peaks were centered at ~385 nm and ~392 nm, respectively (Fig. 3i). However, N-IgM and N-IgG couplings were centered at ~390 nm (Fig. 3j).

### Conjugate pad design, characterization, and activation

**Fig. 4:**
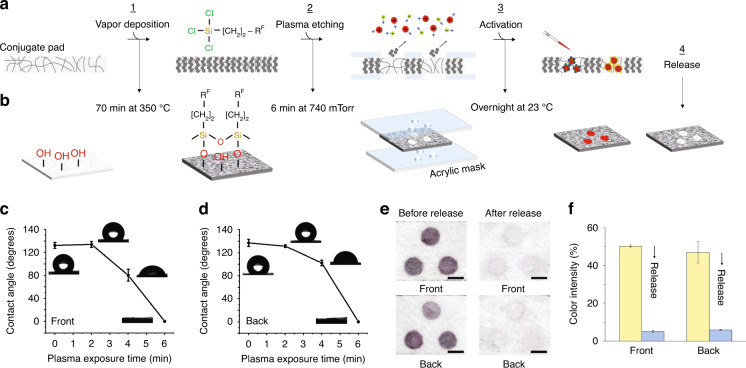
Design, characterization, and activation of the conjugate pad. **a**, **b** Schematics demonstrate the process of generating hydrophobic glass fiber surfaces using PFTS molecules (step 1), selectively removing PFTS molecules using an acrylic mask with 3 holes (~3 mm diameter each) on both sides and plasma etching (step 2), activation of hydrophilic spots with AuNP bioconjugates (step 3) and their release upon rehydration (step 4). **c**, **d** Effect of plasma exposure time on silane desorption, as measured by the water contact angle for the front and back sides. Insets: Respective optical images. Values and error bars: mean ± SD (*n* = 3). **e**, **f** Optical images visually and quantitatively demonstrate the efficient release of AuNP bioconjugates from the hydrophilic conjugate spots upon rehydration. Scale bars: 3 mm. Values and error bars: mean ± SEM (*n* = 3)

The conjugate pad is the upfront component of the HFA, where functionalized AuNPs are kept dry until the assay is performed to specifically capture target analytes. After conjugation, it is crucial to achieve an improved spatial and temporal release of rehydrated AuNPs to enable the flow of analyte-AuNP complexes toward the detection zone of the nitrocellulose membrane without the need for additional control measures. Therefore, the conjugate pad should ideally preserve the functionality of dried AuNPs while exhibiting low binding toward them. It is equally important that the release of analyte-AuNP complexes should minimize cross-reactivity in the detection zone to achieve higher sensitivity. To address these requirements, our third development step involved creating 3 hydrophilic spots (~3 mm diameter each) within the conjugate pad to compartmentalize AuNP-IgE (control), AuNP-S, and AuNP-N bioconjugates. Subsequently, the pretreatment conditions for these spots were optimized to ensure their efficient release upon rehydration (Fig. 4).

Glass fiber was chosen as the conjugate pad for this step due to its advantageous properties, including inertness, which ensures uniform and consistent flow characteristics, and the ability to be chemically modified through silanization, which enables effective compartmentalization by etching. With this in hand, hydrophilic spots were created on the conjugate pads following the procedure described in our previous work^40^. As shown schematically in Fig. 4a, b, PFTS molecules were first vapor deposited onto the conjugate pads to create a hydrophobic surface on the glass fibers (step 1). Next, the silanized pads were transferred to an acrylic mask with 3 distinct laser-cut holes (~3 mm diameter each) on both sides (Supplementary Fig. S3a). Oxygen plasma etching was then used to transfer the hole patterns onto the conjugate pads by selectively removing the PFTS molecules from the fiber surfaces (step 2). As a result, the desired 3 hydrophilic spots were formed, each surrounded by hydrophobic barriers. Subsequently, these spots were pretreated for activation with AuNPs (step 3) and their release upon rehydration (step 4).

During the vapor deposition of PFTS, a vacuum is created by suction, which allows the PFTS to evaporate and condense on the glass fiber surfaces. However, the quality of the deposited PFTS layer, including its hydrophobicity and wettability, depends greatly on the silanization time and temperature. In our experiments, a 75 min silanization time at 350 °C was found to be optimal for generating highly hydrophobic glass fibers, as evidenced by water contact angles of 132° ± 5° on the front side and 137° ± 6° on the back sides of the conjugate pads (Fig. 3c, d). Furthermore, exposing the silanized fibers to oxygen plasma revealed good hydrophobic desorption efficiency on the pads. In fact, a 6-min plasma exposure resulted in the complete hydrophilic transformation of the conjugate pads, as the oxidation of PFTS molecules to silanols (Si−O−H)^41^ made both the front and back sides of the pads immediately wettable by added water droplets (Fig. 3c, d).

After forming the hydrophilic spots in silanized conjugate pads, our next experiments focused on their pretreatment to ensure the efficient release of rehydrated AuNPs. To optimize this process, absorbance measurements and SEM images were used as references before and after release. Additionally, visual inspection and quantitative color intensity analysis of spot images in the detection zones were performed. The results in Fig. 4e, f and Supplementary Fig. S3b–d revealed that sequential pretreatment of the hydrophilic glass fibers with 1% (v/v) BSA and 1% (v/v) Tween-20 facilitates over 95% release of dry AuNP-IgE, AuNP-S, and AuNP-N bioconjugates upon buffer exposure. This outcome was comparable to their release from control undried (wet) conjugate pads (Supplementary Fig. S3b). Pretreatment with BSA served to block the nonspecific binding sites on the glass fiber surfaces, while Tween-20 enhanced the release of rehydrated AuNPs by “stripping” them off. Importantly, the hydrophobic barriers surrounding the hydrophilic conjugate spots were effective in preventing interzone mixing of AuNP-IgE, AuNP-S, and AuNP-N bioconjugates (Fig. 4e), thus allowing their complete vertical low into the underlying detection spots (see Fig. 1d).

Interestingly, it has been reported that sequentially pretreating fibers with 0.05% (v/v) Tween-20 and 20% (w/v) sucrose enhances the release of rehydrated antibodies from polyester conjugate pads^42^. This approach was found to generate highly viscous sample streams, which reduce the velocity of vertical flow, thereby increasing the interaction time of diffused antibodies with the membrane beneath the conjugate pad^42^. This increased interaction time of antibodies with the membrane could potentially improve the overall sensitivity in our assay. Moreover, pretreating our conjugate spots with sugars may also help sustain the long-term stability and serological activity of dried S and N proteins^43^. However, further experimentation with this approach is reserved for future studies.

### HFA sensitivity evaluation

**Fig. 5:**
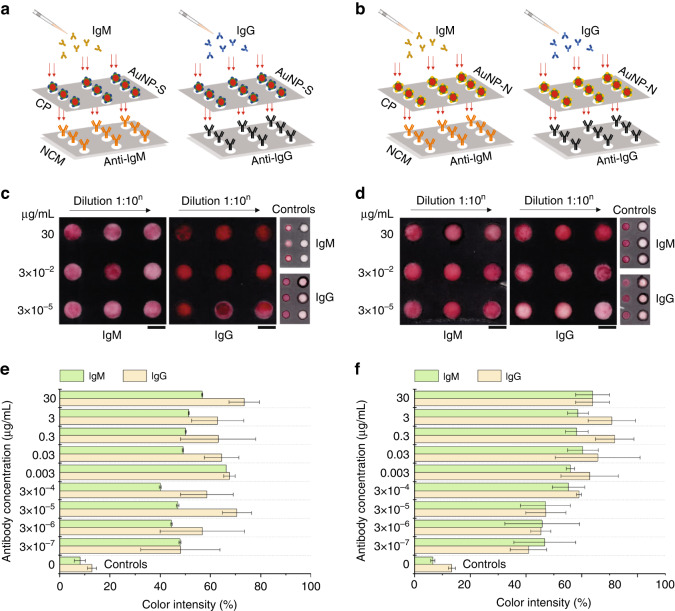
Sensitivity of the HFA. **a**, **b** Schematics represent the experimental setup, where conjugate pads and nitrocellulose membranes (detection layer) are first activated with 3 × 3 spots of dry **a** AuNP-S and **b** AuNP-N bioconjugates and anti-IgM and anti-IgG capture antibodies, respectively. Decreasing concentrations of IgM and IgG antibodies are then directly pipetted onto conjugate spots to interact with AuNP bioconjugates. This is followed by gently pressing the conjugate spots to allow antibody-AuNP complexes to interact with capture antibodies in the nitrocellulose spots. **c**–**f** Overall, the results -respective to experimental procedure described in the schematics of (**a**, **b**)- revealed that compared to the controls, the HFA is highly sensitive toward the capture and detection of IgM and IgG antibodies, providing <1 ng mL^−1^ sensitivity for both (**c**, **e**) AuNP-S bioconjugates and (**d**, **f**) AuNP-N bioconjugates. Direction of the arrows in (**c**, **d**) represents 10-factor serial delutions of IgM and IgG antibodies from left to right and top to bottom, starting with 30 µg mL^-1^. Scale bars: 3 mm. Values and error bars: mean ± SEM (*n* = 3)

The reliability of the HFA directly depends on the specificity and sensitivity that it offers. In the first two development steps, the specificity of the HFA was promoted by functionalizing AuNPs with SARS-CoV-2 S and N proteins, which allowed the selective capture of IgM and IgG antibodies. In the third development step, the conjugate pad design and its pretreatment parameters were optimized for the uniform and consistent release of dry AuNP-S and AuNP-N upon rehydration. With these in hand, the fourth development step focused on evaluating the sensitivity of the HFA (Fig. 5). In the experiments, the conjugate pads and nitrocellulose membranes (i.e., detection layer) were first activated with 3 × 3 spots of dry AuNP-S/N bioconjugates and anti-IgM/IgG capture antibodies, respectively, as previously described. This was followed by pipetting concentrations of 30, 3, 0.3, 0.03, 0.003, 3 × 10^−4^, 3 × 10^−5^, 3 × 10^−6^, and 3 × 10^−7^ µg mL^−1^ IgM and IgG antibodies onto the conjugate pad spots and gently pressing them against the underlying detection spots (Fig. 5a, b).

Another set of experiments was conducted where the same concentrations of IgM and IgG antibodies were allowed to couple with AuNP-S and AuNP-N bioconjugates in their respective buffers. The resulting antibody-AuNP complexes were then pipetted directly onto the nitrocellulose detection spots that were activated with anti-IgM and anti-IgG capture antibodies (Supplementary Fig. S4). The purpose of these experiments was to serve as a control for the sensitivity of antibody-AuNP complexes released from the conjugate pad. In both sets of experiments, the color intensity produced in the back readout of nitrocellulose spots (detection zone) was taken into consideration for visual inspection and quantification.

Furthermore, the successful capture of IgG antibodies in plate wells was confirmed by ELISA testing using anti-IgG antibodies, as shown in Supplementary Fig. S5. The results revealed that the sensitivity of ELISA in detecting IgG antibodies spiked in buffer was comparable to that of the HFA, with a sensitivity of less than 1 ng mL^−1^ for both AuNP-S and AuNP-N bioconjugates. This indicates that HFA offers a fast and convenient alternative while maintaining a high level of sensitivity, similar to traditional ELISA tests commonly used in clinical settings.

Overall, the results shown in Fig. 5c–f demonstrated that HFA is highly sensitive in detecting IgM and IgG antibodies. Excluding the control color intensities in Fig. 5e, f, which read <13% on average, the combined average color intensities for IgM and IgG detection through AuNP-S capture were ~50% ± 3% and 63% ± 10%, respectively. Similarly, for AuNP-N capture, the combined average color intensities for IgM and IgG detection were ~55% ± 7% and 59% ± 7%, respectively. These results supported the previous findings on the specificity of S and N proteins toward IgG and both IgM and IgG, respectively.

Visually, the results further confirmed the uniform and consistent release of antibody-AuNP complexes from the conjugate pads upon rehydration. Surprisingly, however, the control experiments showed that direct detection of AuNP-S-IgM/IgG produced combined average color intensities of ~39% ± 4% and 43% ± 3%, respectively, and those for AuNP-N-IgM/IgG produced ~53% ± 8% and 34% ± 5%, respectively. The reduction in color intensities observed in the experiments was attributed to manual pipetting of the solutions onto the spots. This approach caused variations in the fluid contact angle, which affected the capillary flow rate (i.e., the passive wicking of fluids within the pores of the spots) and introduced shear stresses^33^. Consequently, the flow of the fluid increased, and the interaction time between the antibody-AuNP complexes and the capture antibodies in the spots was reduced, thus leading to a significant decrease in their capture.

Finally, the limit of detection of the HFA was investigated when using healthy plasma to compare the outcomes with those obtained when IgG antibodies were spiked in buffer. In these experiments, the conjugate pad and detection layer of the HFA were activated with dry AuNP-S and AuNP-N bioconjugates and anti-IgG capture antibodies, respectively. Subsequently, we diluted the IgG antibodies in healthy plasma to concentrations of 30, 3, 0.3, 0.03, 0.003, 3 × 10^−4^, 3 × 10^−5^, 3 × 10^−6^, and 3 × 10^−7^ µg mL^−1^ and pipetted them onto the conjugate spots. After gently pressing the conjugate pad, we allowed the AuNP-IgG complexes to interact with the capture antibodies in the detection spots of the HFA. The results obtained from testing the HFA using IgG antibodies spiked in healthy plasma were comparable to those shown in Fig. 5, with a limit of detection of less than 1 ng mL^−1^ (Supplementary Fig. S6). However, the overall color intensity was reduced compared to that obtained when IgG antibodies were spiked into the buffer. We hypothesize that different proteins present in plasma^44^ could potentially compete for binding sites on AuNP-S/N bioconjugates and anti-IgG capture antibodies, thereby interfering with the color intensity observed in the HFA. However, further investigation is needed to confirm this hypothesis.

### HFA functioning and long-term storage stability

**Fig. 6:**
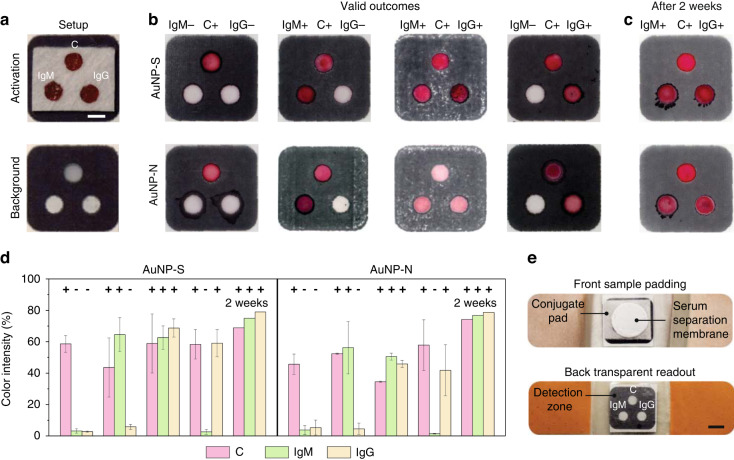
Function and long-term storage stability of HFA. **a** Experimental setup (top panel) shows that the conjugate spots, which are activated with AuNP-IgE (control, C), AuNP-S, and AuNP-N bioconjugates, are carefully aligned with underlying detection spots, which are activated with anti-IgE (control, C), anti-IgM, and anti-IgG capture antibodies for the capture of IgM-AuNP and IgG-AuNP complexes, to ensure efficient functioning of HFA. The bottom panel shows the background spots used as references for color intensity quantification. Prior to the experiments, a plasma/serum separation membrane was placed on top of the conjugate pad. Scale bar: 3 mm. **b** Four different antibody capture and detection conditions (as depicted in Fig. 1c) were tested by pipetting antibody solutions directly onto the plasma/serum separation membrane and gently pressing it afterward. The overall results revealed successful functioning of the HFA with minimal cross-reactivity, as indicated by the valid outcomes depicted in Fig. 1c. **c** The HFA also demonstrated excellent reproducibility for long-term (2 weeks) storage stability. **d** The overall performance of all valid outcomes confirmed whether the sample was negative (IgM−/IgG−) or positive (IgM+/IgG−, IgM+/IgG+, IgM−/IgG+) for SARS‐CoV‐2 infection. Values and error bars: mean ± SEM (*n* = 3). **e** The layers of HFA were combined onto a commercially available adhesive bandage, mimicking the respective schematic in Fig. 1a. Scale bar: 3 mm

After successfully preparing reliable and reproducible conjugate spots that uniformly release antibody-AuNP complexes and detection spots that capture antibodies efficiently and discriminately, the fifth and final development step involved investigating the overall functioning and long-term storage stability of the HFA. This was done by placing the conjugate pad, which was activated with dry AuNP-IgE (control), AuNP-S, and AuNP-N bioconjugates, on top of a nitrocellulose membrane that was activated with dry anti-IgE (control), anti-IgM, and anti-IgG capture antibodies for the capture of IgM-AuNP and IgG-AuNP complexes. To ensure controlled vertical fluid flow, the hydrophilic spots on the conjugate pad were carefully aligned with the corresponding hydrophilic spots on the nitrocellulose membrane (Fig. 6a). Four different antibody capture and detection conditions were then inspected, which included no spiked IgM and IgG (IgM−/IgG−), spiked IgM and no IgG (IgM+/IgG−), spiked IgM and IgG (IgM+/IgG+), and no IgM and spiked IgG (IgM−/IgG+), where the spiked antibody concentrations remained the same for all cases (i.e., 3 µg mL^−1^). To mimic their collective presence in plasma/serum, IgM and IgG antibodies were further mixed at equal concentrations during the IgM+/IgG+ investigation.

In all tested conditions, ~15 µL of antibody solution was directly pipetted onto the plasma separation membrane, and then the membrane was gently pressed against the conjugate pad beneath it (see Fig. 1d). The antibodies were allowed to interact with rehydrated AuNP bioconjugates in the conjugate spots. Immediate color production took place within 3 min after the flow of antibody-AuNP complexes into the underlying detection spots, reaching its optimum by ~15 min. Overall, the results in Fig. 6b, d demonstrated successful functioning of the HFA, both visually and quantitatively. Accordingly, in all valid results with positive outcomes (see Fig. 1c), the presence of spiked IgM and/or IgG antibodies was clearly evident, with average color production ranging from ~35% to 60% in their respective detection spots. Importantly, the overall cross-reactivity of spiked antibodies outside designated detection spots was minimal, with <6% average color intensity production relative to background, as shown in Fig. 6a.

Our next focus was to investigate the long-term storage stability of HFAs. For this purpose, activated HFAs were first stored for 2 weeks in a desiccator. Afterward, IgM and IgG antibodies, spiked at equal concentrations (3 µg mL^−1^) in buffer, were pipetted onto conjugate spots and allowed to produce color in the nitrocellulose detection spots. The results shown in Fig. 6c, d demonstrate that the HFAs exhibit excellent reproducibility for long-term storage stability of both AuNP-S and AuNP-N bioconjugates, as evidenced by no loss of color in the detection spots (~75–78% color intensities for IgM and IgG antibodies).

It is worth noting that during the overall functioning of HFA, antibodies tend to laterally slip in the conjugate pad due to the highly hydrophobic surfaces of the glass fibers^45^. However, the pressure applied by the finger collapses the porous network of fibers, resulting in decreased porosity and permeability^46^. This collapse of fibers directs the lateral flow of antibodies toward the three hydrophilic conjugate spots. Importantly, as the applied pressure increases, the capillary flow rate of antibodies within the conjugate/detection spots decreases^47^. This, in turn, increases the time needed for the remaining dried volume within the spots to passively wick, allowing more time for antibodies to interact with rehydrated AuNPs and capture antibodies in the conjugate and detection spots, respectively. Accordingly, to minimize the ~25% variation in color intensity across the detection spots of the HFA, it will be essential to quantitatively study the decreased permeability in relation to the decreasing porosity of the conjugate pad and nitrocellulose membrane under applied pressure and explore additional means for the efficient delivery of antibodies into the conjugate spots, such as treating the surrounding hydrophobic glass fiber surfaces with various surfactants. These investigations, however, are left for future studies.

## Discussion

In this work, we have developed a novel HFA for the enhanced detection of SARS-CoV-2 IgM and IgG antibodies in blood. The bioactive layers of the HFA were designed to function efficiently, resulting in a visibly distinguishable sensitivity of <1 ng mL^−1^ for the capture of IgM and IgG antibodies spiked both in buffer. This sensitivity was found to be comparable to that achieved when IgG antibodies were spiked in healthy plasma. It should be noted that testing the HFA in buffer is simpler, as it contains only spiked IgM and/or IgG antibodies that can interact with the assay. In plasma, on the other hand, there are numerous other proteins^44^, including human serum albumin, that can compete for binding sites despite treating the HFA with BSA and Tween-20, thus interfering with the color intensity observed in the HFA compared to buffer conditions.

Nevertheless, overall, sensitivity was achieved through the highly specific capture of IgM and IgG antibodies using AuNP-S/N bioconjugates in the conjugate spots. Additionally, over 95% release of antibody-AuNP complexes into the underlying detection spots further contributed to the enhanced sensitivity. Importantly, HFA offers the advantage of being a fast and convenient diagnostic approach while maintaining sensitivity and specificity comparable to those of traditional ELISA tests commonly used in clinical settings. The ELISA method is widely accepted and established as a reliable technique for detecting and measuring specific antibodies in biological samples. Therefore, obtaining results similar to those of the ELISA approach provides confidence in the accuracy and reliability of the HFA. Furthermore, a key feature of the HFA is the direct spot-to-spot vertical fluid flow, which improves the interaction probability between the antibody-AuNP complexes and captures antibodies in the detection spots due to gravity and better capillary action. This vertical fluid flow also allows the capture of high concentrations of target antibodies, thus reducing the assay time to minutes and minimizing the hook effect. Combined, the above characteristics validate the potential of our bandage-based HFA as a promising technology for point-of-care diagnostics, offering a convenient and efficient alternative without compromising accuracy and reliability.

The HFA also showed that S proteins were more reactive in capturing IgG antibodies than IgM antibodies, while the reactivity toward N proteins was similar for both antibodies. Notably, unlike symptomatic individuals, asymptomatic individuals have been reported to exhibit dominant IgG responses against the S1 subunit of the S protein as opposed to IgM responses^48^. Additionally, the S1 subunit purified from mammalian cells has been shown to have the highest performance in distinguishing COVID-19 patients from controls^49^. N protein-based antibody assays, on the other hand, have been reported to have higher false-negative rates compared to the S1 subunit, and antibody titers against N proteins decline significantly after 16 weeks post-infection^50^. Thus, to make HFA testing more reliable and efficient, these findings can be used as a reference, especially when targeting the capture of dynamically produced IgM and IgG antibodies in asymptomatic individuals. Based on our specificity results, we hypothesize that conjugating AuNPs in the HFA solely with S proteins would result in higher positive predictive values for SARS-CoV-2 infection (i.e., the probability of actually having the infection when testing positive) for both symptomatic and asymptomatic individuals. However, further investigation into specificity using clinical blood samples that do and do not contain (control) IgM and IgG is necessary for the complete implementation of our approach. This is left for future studies.

In general, antibody tests are considered based on the sensitivity and specificity that they offer. For tests with diagnostic/efficacy ability, higher sensitivity (i.e., fewer false-negative results) is beneficial for accurately identifying infected individuals. Higher specificity (i.e., fewer false-positive results) is important for accurately identifying noninfected individuals. In this regard, the HFA demonstrates promising diagnostic/efficacy capabilities in terms of sensitivity and specificity. Moreover, compared to other similarly developed VFAs^19,26^, the HFA has a simpler design and function, as it does not require additional flow-through films or origami-style multilayers for uniform and controlled delivery of analyte-AuNP complexes^51,52^. Instead, the HFA utilizes the nitrocellulose membrane for direct detection of targeted analytes using covalently bonded detection AuNPs, which offers a viable alternative route. However, in HFA and in VFAs in general, finger pricking needs to be done separately using lancets and may pose a risk of contamination. Therefore, an important improvement aspect for the HFA would be to incorporate biodegradable porous microneedles^53^ into the front sample padding, enabling efficient finger pricking via in situ puncturing. This would eliminate the need for lancets, thus reducing the extra step for obtaining a blood sample and minimizing the risks of infection at the puncture site. However, further investigation of the efficiency of plasma/serum separation under various factors, such as the presence of clotting factor proteins in clinical samples, along with incorporating microneedles into the HFA, is left for future studies.

The major strengths of point-of-care antibody tests lie in their ability to scale up production to meet market demand while reducing production costs. These tests are also designated to be user-friendly with simple and rapid one-step results that do not require any training or expert interpretation. Thus, the design of the HFA was inspired by the need for scalability for mass testing and broad use by the general population. As shown in Fig. 6e, the HFA layers were combined onto a commercially available adhesive bandage, resembling the schematic in Fig. 1a. These bandages can be manufactured in batches (Supplementary Fig. S7), shipped, and stored at room temperature, allowing at-home testing in households. This approach will enable rapid and sensitive detection of the immune response to SARS-CoV-2. In the future, the bandage can also be used for real-time screening of virus spread by involving smartphones or portable readers in at-home diagnosis approaches. Measuring the test results across the population using this technology will have a tremendously positive impact in various settings, such as hospitals, schools, airports, factories, and other public facilities and rural regions. as the timely identification of infected individuals is the first line of defense against viral attacks and spread. Moreover, due to its simple design, practicality, and functionality, this technology will also hold promise for inexpensive, robust, accurate, rapid, quantitative, and minimally invasive testing in various settings.

The technology has the potential to enable various other antibody tests to be performed quickly and efficiently in identical settings, with results available within minutes. This can be achieved by optimizing its AuNPs and detection layer (readout) with appropriate antigens and capturing antibodies related to any future pandemics or outbreaks. Here, the choice of antigens will be based on capturing the corresponding protective antibodies that are produced in the blood during and after viral or bacterial infections. In this way, the technology will allow screening of the immune response of the entire population, enabling a more comprehensive investigation of pandemics. Importantly, the benefits of using an adhesive bandage format, as described above, are expected to contribute to the growth of the global serology-based adhesive bandage market in the future.

## Conclusions

With the COVID-19, the urgent need for effective point-of-care antibody tests became apparent, especially the ones that can assess the early immune response to infections. Unlike tests that look for pathogenic genetic material in the throat or nasal swabs, antibody tests detect the presence of IgM and/or IgG antibodies in the blood. These antibodies are practical biomarkers for the diagnosis and severity prediction of infections in both symptomatic and asymptomatic individuals. Through the development of the novel HFA, we have demonstrated that an affordable point-of-care antibody test device can be constructed in the form of an adhesive bandage. With its established specificity, sensitivity, reproducibility, and long-term storage stability, the adhesive bandage format does not require any training or expert judgment for the interpretation of the results. It can be manufactured at high volume and low cost and can be easily modified to perform various other antibody tests in one step by simply modifying its conjugate pad and detection zone with multiple (>3) different AuNP bioconjugates and capture antibodies specific to any pathogenic disease, whether viral or bacterial. The adhesive bandage format also allows rapid detection of the immune response to pathogen infections within minutes and enables real-time monitoring of their spread. As a result, conducting prospective tests with the bandage can be useful in estimating the accurate burden of pandemics and, importantly, in reducing the number of new cases from one day to another, thus helping to “flatten the curve” by taking preventative measures.

## Materials and methods

### Materials

Carboxylic acid-functionalized gold nanoparticles (AuNPs) (40 nm diameter) were obtained from nanoComposix. Whatman standard fiberglass conjugate pads (0.3 mm thickness) and filter paper (~6 μm pore size) were obtained from Cytiva. Vivid 120 nitrocellulose membrane (0.2 mm thickness) was obtained from Pall Europe. 1-Ethyl-3-(3-dimethylaminopropyl) carbodiimide hydrochloride (EDC), N-hydroxysulfosuccinimide (NHS), and protein-free blocking buffer (Pierce) were obtained from ThermoFisher Scientific. Bovine serum albumin (BSA), human polyclonal goat anti-IgM (I0759) and anti-IgG (I2136) secondary (capture) antibodies, Trizma base, and trichloro(1H,1H,2H,2H-perfluorooctyl) silane (PFTS) were obtained from Sigma‒Aldrich. SARS-CoV-2 S (REC31806) and N (REC31851) proteins, human recombinant IgE (his-tag) protein (REC31705-100), anti-IgE secondary (capture) antibodies, human monoclonal IgG1 anti-SARS-CoV-2 S1 (MAB12422-100), IgG1 anti-SARS-CoV-2 N (MAB12437-100), IgM anti-SARS-CoV-2 S (MAB12423-50), and IgM anti-SARS-CoV-2 N (MAB12438-50) were obtained from Native Antigen Company.

### Blood collection

A blood sample was collected from a healthy donor and placed in a tube containing EDTA. The sample was then processed using the plasma/serum separation membrane (filter paper) of the HFA to assess the efficiency of the membrane in separating the plasma/serum. The experiments were conducted in accordance with the approved NYU Abu Dhabi Institutional Review Board (IRB) protocols.

### Plasma recovery

Plasma quality was measured following the protocol described in ref. ^54^. Briefly, a plate reader (Thermo Fisher) was used to measure the concentration of hemoglobin in plasma samples separated using the filter paper of the HFA or centrifugation (control). The plasma separation efficiency was then calculated using:

1 $${Efficiency}=\{1-[{Abs}\left({Recovered}\,{Plasma}\right)-{Abs}\left({Blood}\right)-{Abs}\left({Centrifuged}\,{Plasma}\right)\}* 100$$

where Abs (Recovered Plasma), Abs (Blood), and Abs (Centrifuged Plasma) are respective absorbance values as measured from optical densities at 412 nm, 407 nm, and 575 nm. Here, the concentration of red blood cells in the recovered plasma, determined as the background signal at 575 nm, was subtracted from the hemoglobin concentration in the recovered plasma. The concentration of red blood cells in the centrifuged plasma was negligible.

### AuNP conjugation

The covalent conjugation of SARS-CoV-2 S and N proteins to carboxylated AuNPs was carried out using EDC/NHS chemistry. Prior to conjugation, 1 mL MES buffer at pH 6 was added to 200 µL carboxylated AuNPs in 2 separate Eppendorf tubes: one for S protein conjugation and the other for N protein conjugation. Solutions of EDC and NHS were prepared in deionized water at concentrations of 2 mg mL^−1^ and 10 mg mL^−1^, respectively. Then, 20 µL of EDC was added to the AuNP solutions and incubated for 10 min in the dark, followed by the addition of 8 µL of NHS. The Eppendorf tubes were then covered with aluminum foil, placed on a rocker, and incubated for 20 min at 95 motions min^−1^. Subsequently, the AuNP-NHS solutions were washed twice by centrifuging AuNP-NHS complexes in a 5424 R microcentrifuge (Eppendorf) for 10 min at 15,000 rcf and 10 °C. The supernatant was removed, and the pellet was resuspended in 1 mL of disodium phosphate solution at pH 7.5 for S protein conjugation and in potassium carbonate solution at pH 11 for N protein conjugations. From 1 mg mL^−1^ protein solutions, 10 µL of S and N proteins were added to the respective tubes and incubated for 90 min on a rocker at 95 motions min^−1^ to allow assembly of AuNP-S and AuNP-N bioconjugates. Afterward, the solutions were blocked with 0.5% (v/v) protein-free blocking buffer for 30 min and centrifuged at 15,000 rcf for 10 min at 10 °C. The supernatant was discarded, and the pellets were resuspended in 200 µL solutions of potassium carbonate or disodium phosphate. An additional 20 µL of Trizma base was added to the AuNP-N solution for increased stability. Control AuNP-IgE bioconjugates were prepared similarly using 10 µg mL^−1^ IgE protein.

AuNP bioconjugates were characterized using a UV-2700 spectrophotometer (Shimadzu) to record changes in the absorbance spectra of AuNPs before and after each step of functionalization. Diluted solutions of AuNP-S and AuNP-N at 200 µL were brought up to 1 mL in a quartz cuvette (Thermo Fisher Scientific) using deionized water and placed in the spectrophotometer for measurements. AuNP-IgE controls were also recorded at a concentration of 5 µg mL^−1^. Fluorescence intensities for AuNP-S and AuNP-N, as well as their coupling with SARS-CoV-2 IgG and IgM antibodies, were recorded using a Fluoromax-4 spectrophotometer (Horiba). Fluorescence was measured using 20 µL of AuNP-S/N and 3 µL of IgG/IgM at concentrations of 1 mg mL^−1^. The solutions were then diluted to 1255 µL with 600 µL of deionized water, 625 µL of buffer, and 20 µL of AuNP-S/N composite.

### TEM and SEM imaging

The functionalized AuNPs were further examined for any changes in size or behavior using a Talos F200X transmission electron microscope (ThermoFisher). To prepare for imaging, the AuNP solutions were diluted to a 1:20 ratio, deposited onto microscopic grids, and then left to dry overnight. In addition, the conjugate pads were imaged before and after releasing the AuNPs using a Quanta3D Scanning Electron Microscope (ThermoFisher) at a voltage of 5 keV and a spot size of 2.0. Prior to imaging, conjugate pads were coated with a layer of gold ~10 nm thick using a Cressington 108 Auto Sputter Coater. Conjugate pads of similar size (1 cm^2^) were loaded with either AuNP-S/N bioconjugate and allowed to dry. Another set of loaded pads, representing the released AuNP conjugates, were suspended in a buffer to release the AuNP conjugates, dried, and gold-coated before SEM imaging. The images were then acquired digitally using UltraScan software (UltraScan Project).

### HFA layers

The HFA consists of three distinct layers stacked vertically (see Fig. 1c). The first layer is the plasma separation membrane, which was cut into circular pieces with a diameter of ~1 cm. The second layer is the glass fiber conjugate pad, which was cut into ~1 × 1 cm^2^ pieces and vapor-treated with 600 µL of PFTS molecules to create hydrophobic surfaces on the glass fibers. The treatment was performed in a desiccator for 75 min at 350 °C. The treated (silanized) conjugate pads were then placed in an acrylic mask with 3 laser-cut holes, each with a diameter of ~3 mm, on both sides. The hydrophilic spots on the conjugate pad were then created by placing the mask in an oxygen plasma cleaner (Harrick Plasma) for 6 min at 740 mTorr, after which they were pretreated with 1% (v/v) BSA and 1% (v/v) Tween-20. The modified conjugate pads were then dried in a desiccator for 2 h. Next, the corresponding spots on the pads were loaded with 4.5 µL of AuNP-IgE (control), AuNP-S, and AuNP-N solutions and left to dry in the desiccator overnight. The investigation of the release efficiency of AuNPs upon rehydration was carried out by suspending and gently shaking the pads in their respective buffers. The absorbance spectra of the released AuNPs were measured using a UV-2700 spectrophotometer (Shimadzu).

The third layer is the nitrocellulose membrane, which was decorated with a 3-spot pattern (each with a diameter of ~3 mm) using a ColorQube 8570 wax printer (Xerox). The spots were designed to match the pattern of the hydrophilic spots in the conjugate pad. Next, the nitrocellulose membranes were placed in an oven for 7 min at 85 °C for the wax to diffuse and create the desired 3D hydrophobicity around and within the spots. Next, 2 µL of anti-IgE, anti-IgG, and anti-IgM capture antibodies, diluted to concentrations of 1 mg mL^−1^ in buffer, were pipetted onto their corresponding spots on the nitrocellulose membranes and allowed to physically adsorb onto the fibers for 30 min. After drying, 0.05% (v/v) Tween-20 was added into each spot to prevent any nonspecific adsorption. The modified conjugate pads and nitrocellulose membranes were then placed in sealed Petri dishes and kept in a desiccator until further use.

### Antibody concentration measurements

SARS-CoV-2 anti-S-IgM, anti-S-IgG, anti-N-IgM, and anti-N-IgG antibodies were first diluted in buffer to concentrations of 30, 3, 0.3, 0.03, 0.003, 3 × 10^−4^, 3 × 10^−5^, 3 × 10^−6^ and 3 × 10^−7 ^µg mL^−1^. Separately, 2 µL of anti-IgM and anti-IgG capture antibodies at concentrations of 3 µg mL^−1^ were loaded on nitrocellulose membranes in a 3 × 3 pattern. For the control sets, one line of 3 spots was treated with 2 µL of anti-IgM/anti-IgG capture antibodies at concentrations of 3 µg mL^−1^, while the other line of 3 spots was left untreated. All spots were then blocked using 0.5% (v/v) protein-free blocking buffer and 0.05% (v/v) Tween-20. Subsequently, 18 µL of AuNP-IgE (control) and AuNP-S/N solutions were loaded into conjugate pads, which were pretreated with 1% (v/v) BSA and 1% (v/v) Tween-20 and had sizes matching the spots on the nitrocellulose membrane. The conjugate pads were then assembled on top of the nitrocellulose membrane spots, and the assemblies were placed in sealed Petri dishes and stored overnight in a desiccator. Next, antibody concentration measurements were performed by pipetting 4.5 µL from each of the IgG and IgM concentrations onto the corresponding conjugate pad spots, followed by gently pressing the conjugate spots against the underlying detection spots. The plasma separation membrane was excluded during the experiments.

The validation of the binding of serially diluted IgG antibodies onto plate wells was performed using an ELISA test at 23 °C. For this purpose, the surface of the well plates was first covered with silane molecules containing 0.5% (wt) 3-aminopropyltriethoxysilane (APTES, Sigma‒Aldrich), following the method described in our previous work^55^. Next, anti-IgG capture antibodies were immobilized on the salinized surfaces for 45 min. IgG antibodies, serially diluted in buffer to concentrations of 30, 3, 0.3, 0.03, 0.003, 3 × 10^−4^, 3 × 10^−5^, 3 × 10^−6^ and 3 × 10^−7 ^µg mL^−1^, were then spotted onto the wells and allowed to interact with anti-IgG capture antibodies for 60 min. After washing and blocking steps with buffer and BSA, respectively, mouse IgG antibodies tagged with FITC were added to the wells to interact with IgG antibodies for 60 min. The wells were then washed with buffer, after which they were transferred to a plate reader (ThermoFisher) for optical density measurements. For each set, detection IgG antibodies were also incubated on silanized surfaces at equal concentrations to serve as controls for the background signal.

### HFA performance

The nitrocellulose membranes were scanned from both the front sample and back readout sides using a Perfection V850 Pro photo scanner (Epson). The acquired images were then quantified for color intensity using ImageJ software (https://imagej.net). First, the intensity of pixels was measured as gray values, with ranges of 0 (pure black) to 255 (pure white). Next, the data were normalized to obtain % color intensity^56^, as shown in Eq. 2.

2 $${\rm{Color}}\,{\rm{intensity}}=100-\left(100\times {\rm{measured}}\,{\rm{gray}}\,{\rm{value}}\right)/255$$

For the control spots, the color intensity was determined by subtracting the measured color intensity from the background (i.e., no modification) and recording the result.

### Statistical analysis

The values are presented as the mean ± standard error of the mean (SEM) and were obtained from at least three repeats per experiment. Plots were generated using OriginPro data analysis and graphing software (OriginLab). Where appropriate, statistical analysis was conducted with OriginPro using a two-sample *t* test. A *p* value of <0.05 was considered statistically significant.

## Supplementary information

Supplementary Information
