# Supplementary material for: Spike- and nucleocapsid-based gold colloid assay toward the development of an adhesive bandage for rapid SARS-CoV-2 immune response detection and screening
Source: Microsyst Nanoeng. 2023 Jun 20;9:82. doi: 10.1038/s41378-023-00554-8 (PMC10281977; doi:10.1038/s41378-023-00554-8)
Supplement: Supplementary file 1 — Supplementary Information [file 41378_2023_554_MOESM1_ESM.docx]

**Spike- and nucleocapsid-based gold colloid assay toward the development of an adhesive bandage for rapid SARS-CoV-2 immune response detection and screening**

Imen Boumar ^a, 1^, Muhammedin Deliorman ^a, 1^, Pavithra Sukumar ^a^, and Mohammad A. Qasaimeh ^a, b, *^

*^a^ Division of Engineering, New York University Abu Dhabi (NYUAD), UAE*

*^b^ Department of Mechanical and Aerospace Engineering, New York University, USA*

*^1^ These authors contributed equally as co-first authors.*

*^*^ Corresponding author. E-mail address:* [*mohammad.qasaimeh@nyu.edu*](mailto:mohammad.qasaimeh@nyu.edu)*.*


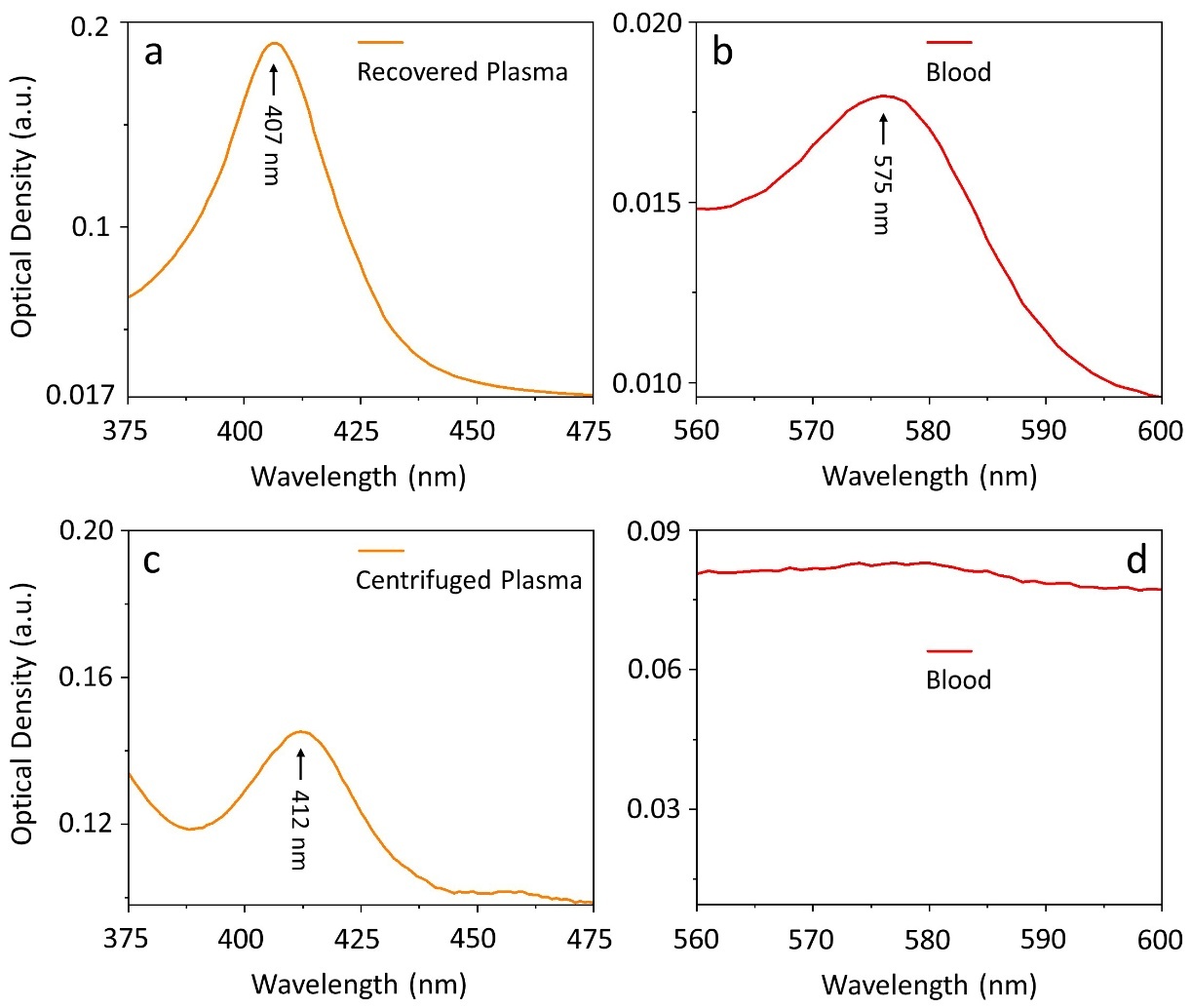


**Fig. S1. UV-Vis spectroscopy analysis of recovered plasma and control centrifuged plasma. (a)** The concentration of hemoglobin in the recovered plasma measured at 407 nm. **(b)** The concentration of red blood cells in the recovered plasma measured at 575 nm. **(c)** The concentration of hemoglobin in the control centrifuged plasma measured at 412 nm. **(d)** The concentration of red blood cells in the control centrifuged plasma was negligible.


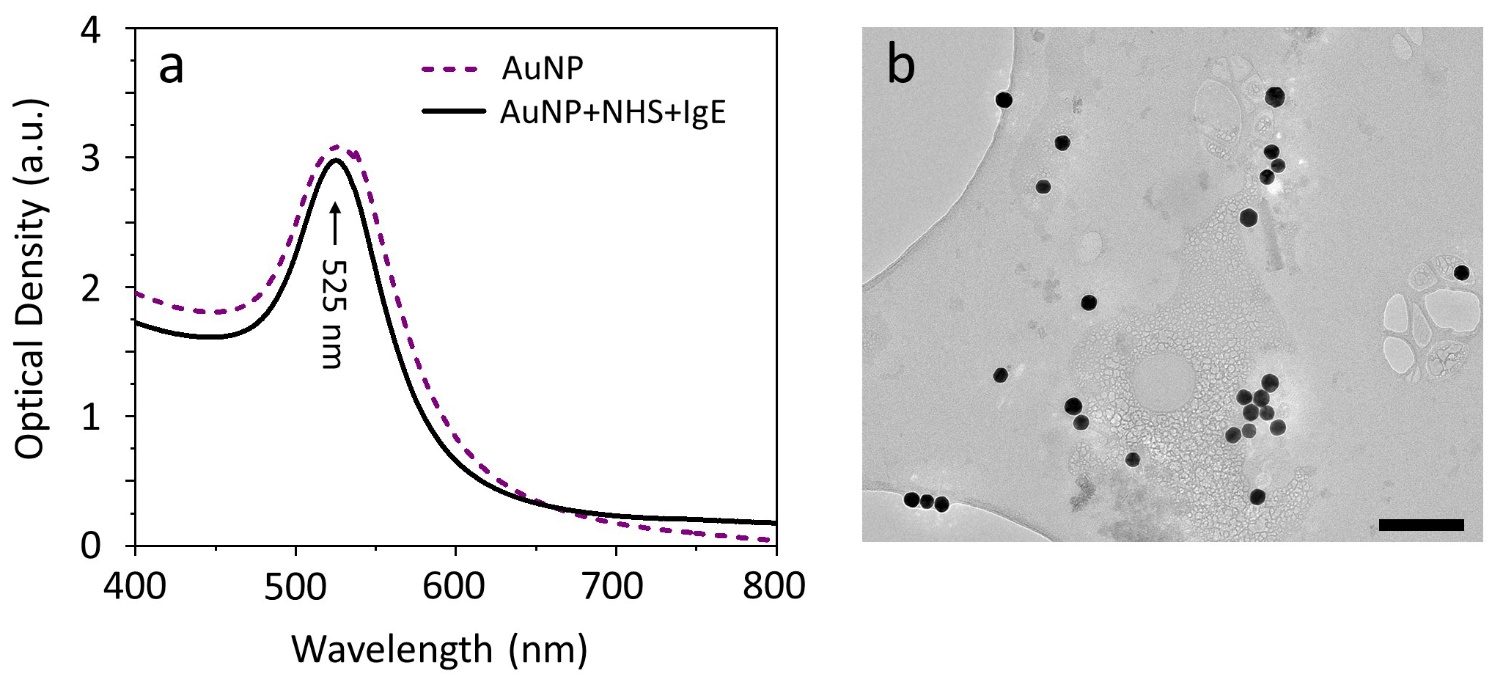


**Fig. S2. Bioactivation of AuNPs with IgE protein and their characterization.** **(a,b)** Optical density measurements and TEM images confirm stable (monodispersed) solutions after chemisorption of each functional group to each layer of activated AuNP surfaces. Scale bar: 200 nm.


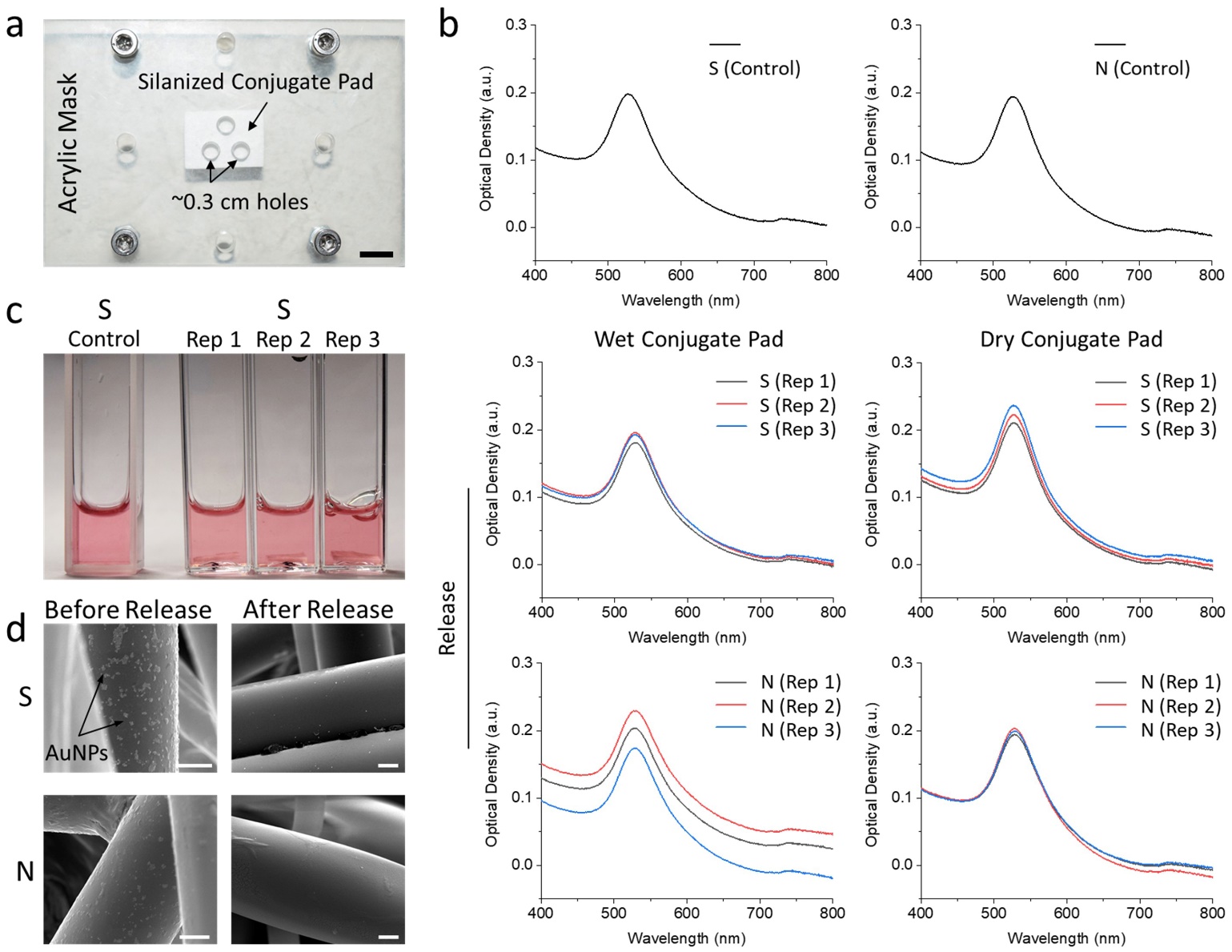


**Fig. S3. The acrylic mask and characterization of the release of AuNPs upon rehydration.** **(a)** Micrograph shows the acrylic mask with a silanized conjugate pad sandwiched between its identical front and back parts. The 3 distinct laser-cut holes (~3 mm diameter each) on both sides of the mask were used to transfer the spot pattern onto the conjugate pad via plasma etching. Scale bar: 6 mm. **(b,c)** Optical density measurements and example micrographs show the released AuNP-S and AuNP-N bioconjugates from undried (wet, control) and dried conjugate spots upon buffer exposure. **(d)** Example SEM images of AuNPs before and after their release from conjugate spots. Scale bars: 1 μm.


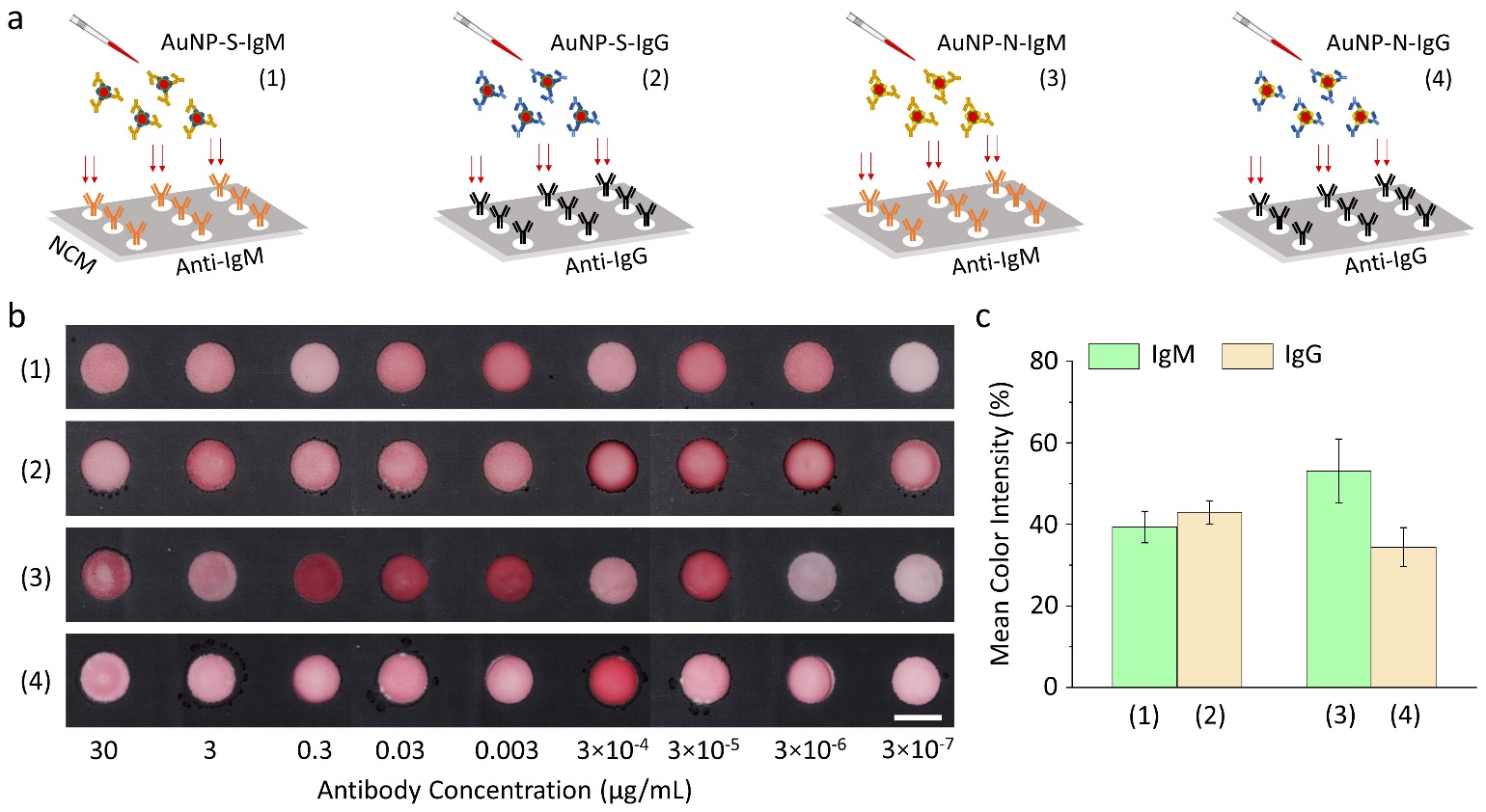


**Fig. S4. Sensitivity of IgM and IgG antibodies directly coupled with AuNP-S and AuNP-N bioconjugates in their respective buffers.** **(a)** Schematic represents the experimental setup, where the resulting antibody-AuNP complexes were pipetted directly onto the nitrocellulose detection spots activated with anti-IgM and anti-IgG capture antibodies. **(b,c)** Overall, results revealed a % reduction in the color intensities compared to results in Fig. 5, which was attributed to manual pipetting of the solutions onto the spots. Scale bar: 3 mm. Values and error bars: mean ± SEM (n = 3).


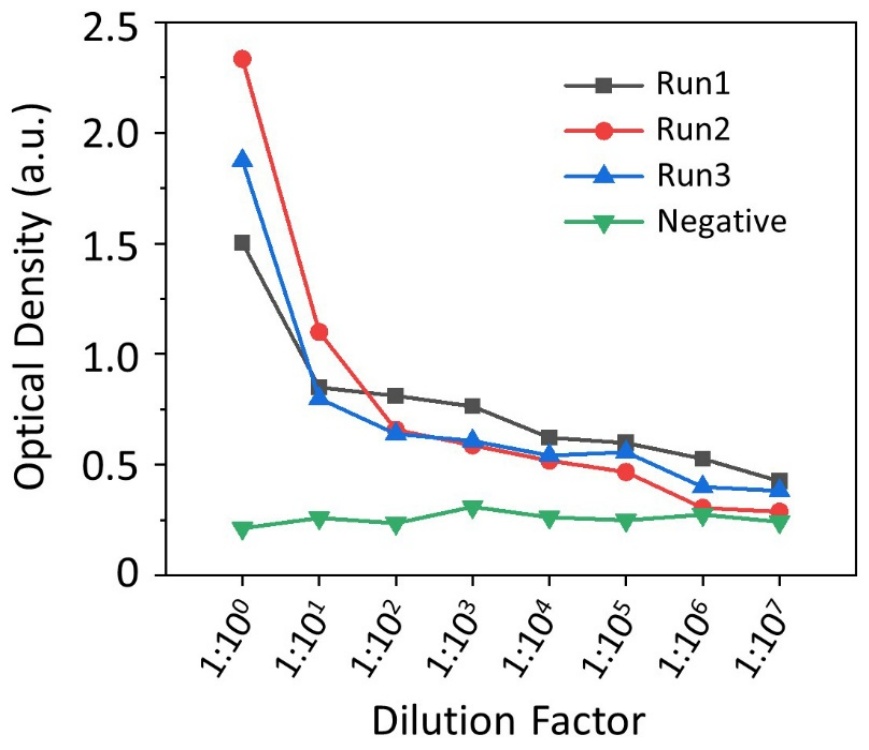


**Fig. S5. Validation of the binding of IgG antibodies using ELISA test.** After washing and blocking steps, a plate reader was used to scan the IgG antibodies (serially diluted in buffer to concentrations of 30, 3, 0.3, 0.03, 0.003, 3 × 10^-4^, 3 × 10^-5^, 3 × 10^-6^ and 3 × 10^-7^ µg·mL^-1^) for binding onto plate wells using anti-IgG secondary (capture) antibodies and detection IgG antibodies tagged with FITC. For each condition, the background signal served as a negative control, where FITC-tagged IgG antibodies were allowed to non-specifically bind onto well plates at equal concentrations.


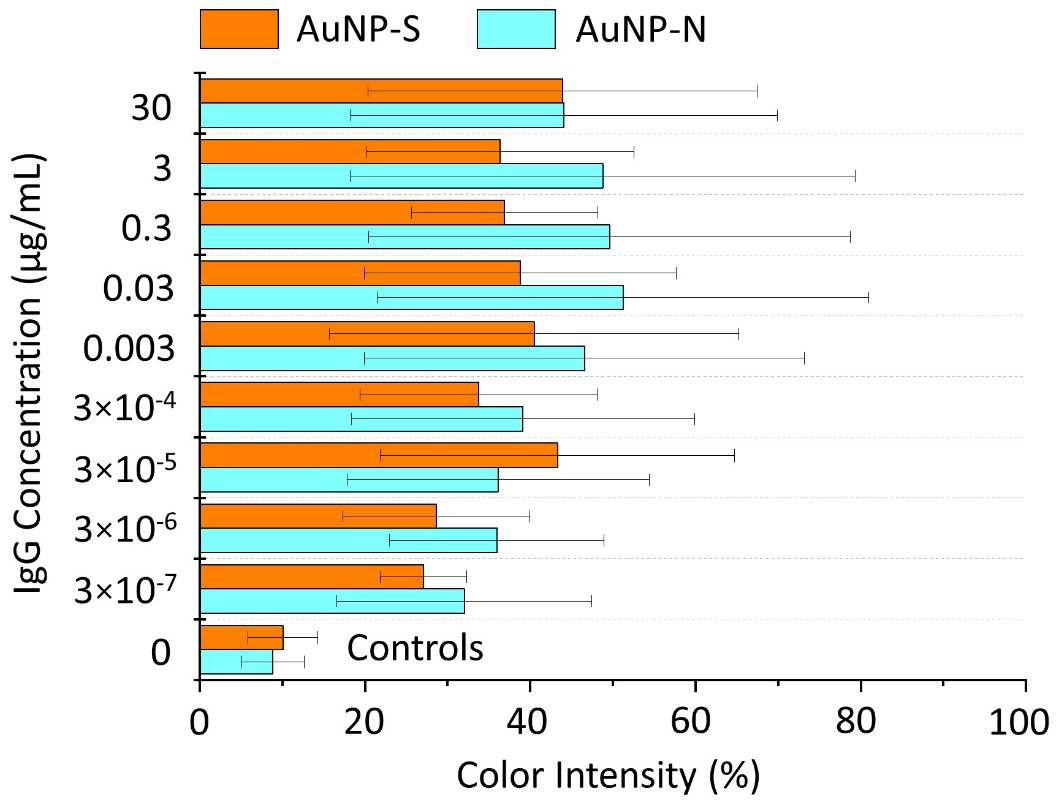


**Fig. S6. Investigation of the sensitivity of the HFA with IgG antibodies spiked in healthy plasma/serum.** Initially, the conjugate pads and detection layer were activated with 3 × 3 spots of dry AuNP-S and AuNP-N bioconjugates, as well as anti-IgG capture antibodies, respectively. Varying concentrations of IgG antibodies spiked in healthy plasma/serum were then directly pipetted onto the conjugate spots and allowed to interact with the AuNP bioconjugates. Subsequently, the conjugate spots were gently pressed to facilitate the interaction of antibody-AuNP complexes with the capture antibodies in the nitrocellulose spots. The results revealed that compared to controls, the developed HFA demonstrated sensitivity towards the capture and detection of IgG antibodies spiked in healthy plasma/serum, providing a sensitivity of <1 ng·mL-1 for both AuNP-S and AuNP-N bioconjugates. Values and error bars: mean ± SEM (n = 3).


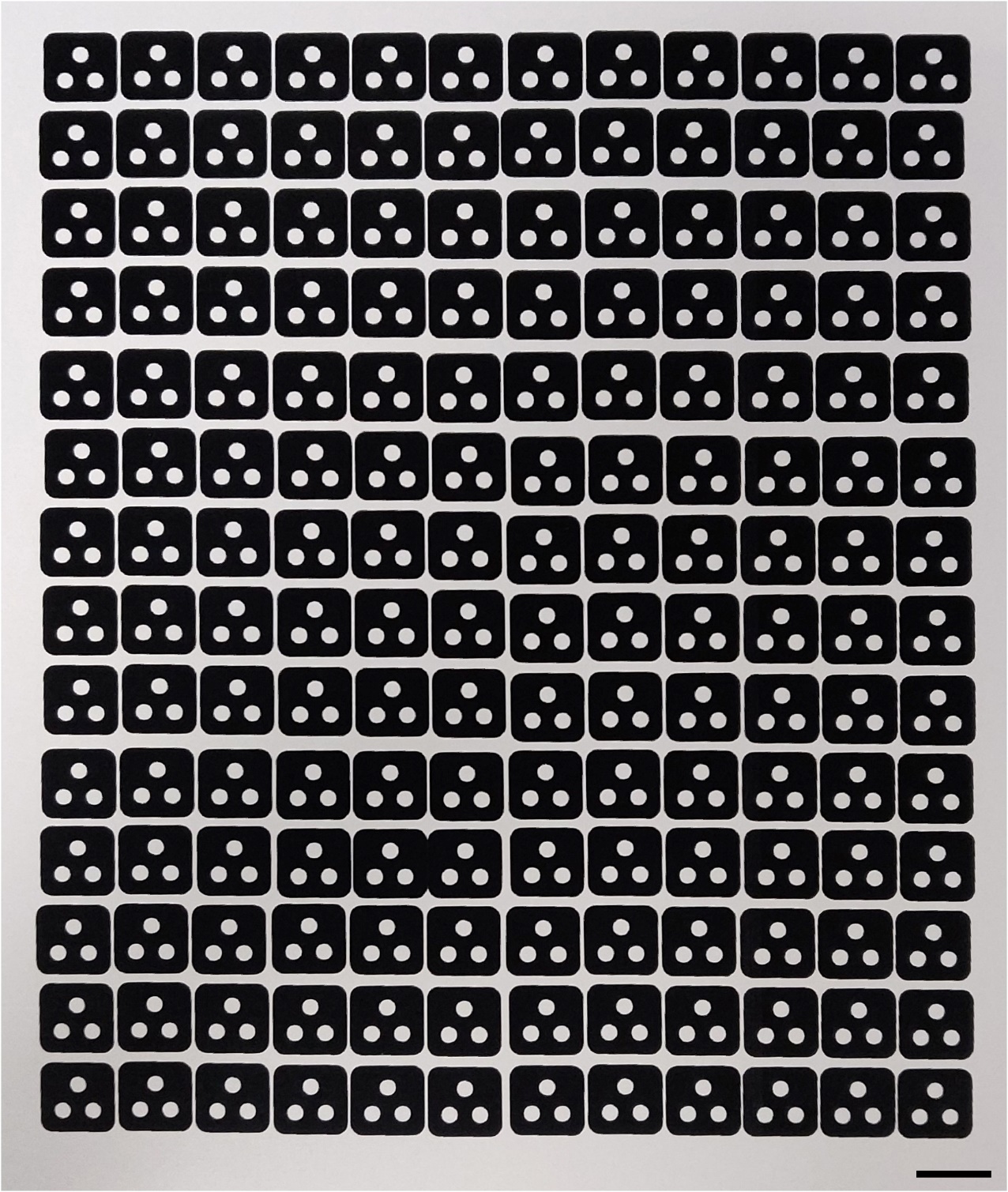


**Fig. S7. Scalability of the HFA.** The micrograph displays the detection zones, consisting of 3 spots, which are wax-printed on A4 paper for batch production. It is worth noting that, depending on the number of targeted analytes, multiple spots (>3) can also be printed accordingly. Scale bar: 16 mm.
